# Silencing TRIP13 inhibits cell growth and metastasis of hepatocellular carcinoma by activating of TGF-β1/smad3

**DOI:** 10.1186/s12935-018-0704-y

**Published:** 2018-12-17

**Authors:** Jianning Yao, Xuexiu Zhang, Jiaheng Li, Dongyao Zhao, Bing Gao, Haining Zhou, Shilin Gao, Lianfeng Zhang

**Affiliations:** 1 Department of Gastroenterology, The First Affiliated Hospital of Zhengzhou University, No.1 East Jianshe Road, Erqi District, Zhengzhou, 450052 Henan China; 2 Reproductive Medicine Department, The Third Affiliated Hospital of Zhengzhou University, No.7 Kangfuqian Road, Erqi District, Zhengzhou, 450052 Henan China

**Keywords:** Bioinformatics, TRIP13, HCC, Metastasis, TGF-β1/smad3

## Abstract

**Background:**

TRIP13 is highly expressed in several cancers and is closely connected with cancer progression. However, its roles on the growth and metastasis of hepatocellular carcinoma (HCC), and the underlying mechanism are still unclear.

**Methods:**

Combining bioinformatics with previous studies, the correlation between TRIP13 and HCC was predicted. TRIP13 expressions from 52 HCC patients and several cell lines were determined. The effects of silencing TRIP13 on cell viability, apoptosis, migration and invasion were respectively detected using CCK-8, flow cytometry and Transwell. qRT-PCR and western blot were performed to reveal associated mechanism. A HCC model was established in BALB/c-nu mice by transplanting HepG2 cells. TRIP13 protein expression and apoptosis in mice tissues were accordingly detected by Immunohistochemistry and TUNEL.

**Results:**

High expression of TRIP13 in HCC affected the survival rate and it was enriched in RNA degradation and fatty acid metabolism according to bioinformatics and prediction from previous literature. Increased expression of TRIP13 in HCC patient tissues was associated with the progression of HCC. Silencing TRIP13 inhibited cell viability, migration and invasion, and induced cell apoptosis. TRIP13 knockdown also suppressed the formation of tumor in vivo. Meanwhile, silencing TRIP13 decreased the expressions of Ki67 and MMP-2 and increased the expressions of TIMP-2, active-caspase-3 and TGF-β1/smad3 signaling- related genes.

**Conclusions:**

Silencing TRIP13 acts as a tumor suppresser of HCC to repress cell growth and metastasis in vitro and in vivo, and such a phenomenon possibly involved activation of TGF-β1/smad3 signaling.

## Background

Liver cancer is the sixth most commonly diagnosed cancer and the fourth leading cause of cancer death worldwide as approximately 841,080 new cases diagnosed and 781,631 deaths in 2018 took place annually [1]. Primary liver cancer includes hepatocellular carcinoma (HCC) (with comprising 75–85% of cases), intrahepatic cholangiocarcinoma and other rare types [1]. About 383,000 new cases of HCC are diagnosed in China annually, accounting for about half of the incidence worldwide, and its mortality rate is also the second highest cause of death among all malignant tumors in China [2]. Most patients with hepatocellular carcinoma have chronic viral hepatitis, especially hepatitis B and C. Among them, hepatitis B is a primary cause in Asia and Africa, while hepatitis C is the main cause in Europe and America [3]. In addition, the risks of non-viral hepatitis including alcoholic and non-alcoholic hepatitis, hereditary hemochromatosis, hepatolenticular degeneration, primary biliary cirrhosis and aflatoxin are increasing [4–6]. Although a clear understanding of the risk factors for carcinogenesis in HCC has been established and the curative effect of early postoperative patients with HCC is effective, the prognosis of HCC is still poor as overall 5-year survival rate remains approximately 50% [7, 8]. An important factor of poor prognosis of liver cancer is the recurrence and metastasis of HCC. About 50% of the patients undergoing radical hepatectomy have occult intrahepatic metastasis or recurrence of residual intrahepatic liver cancer [9].

Metastasis and recurrence of liver cancer is a multistep, multifactorial process, which consists of HCC oncogene activation, tumor suppressor gene inactivation and mismatch repair gene mutation [10–13]. Alternatively known as 16E1BP, TRIP13 is a protein encoded by the TRIP13 gene that interacts with thyroid hormone receptors. TRIP13 is also a member of the AAA^+^ protein family, which can alter the conformation of terminal macromolecules, therefore affecting cell signaling pathways and participating in many cell activities [14–16]. TRIP13 plays an important role in meiosis and mitosis, especially it not only enables chromosome re-pairing and association, but also activates recombination detection points for double-stranded DNA breaks and affects the role of spindle assembly checkpoints [17–22]. TRIP13 is identified as an oncogene, whose overexpression can lead to many human cancers. A large number of studies suggested that TRIP13 gene is highly expressed in head and neck cancer, prostate cancer, lung cancer and breast cancer tissues and is closely associated with colorectal cancer, gastric cancer [16, 21, 23–26].

The possibly mechanism of TRIP13 in the progression of HCC is still poorly understood. To the best of our knowledge, several signaling pathways, for example, Wnt/β-catenin signaling, Hedgehog signaling, AKT signaling and TGF-β signaling are reported to participate in the occurrence and development of liver cancer [27–30]. In this study, we applied bioinformatics to predict the correlation between TRIP13 and HCC, and explore roles of TRIP13 on growth and metastasis of HCC as well as the underlying mechanism.

## Methods

### Screening genes of differential expression in hepatocellular carcinoma

Fifty normal liver samples and 374 cancer tissues of HCC samples were downloaded from The Cancer Genome Atlas (TCGA, https://cancergenome.nih.gov/). The genes of differential expression (DEGs) were analyzed using edgeR and wilcoxTest in combination with survival analysis. We identified DEGs as differentially expressed by |log_2_FC| > 1 and adjusted P value to < 0.05. The visual hierarchical cluster analysis was performed using volcano plot and heat map in ImageGP (http://www.ehbio.com/ImageGP/index.php/Home/Index/index.html) software.

### Gene set enrichment analysis (GSEA)

According to the Kyoto Encyclopedia of Genes and Genomes (KEGG) pathways, GSEA was used to analyze biological significance. Above screened DEGs were applied to conduct enrichment analysis. The expression data of total normalized mRNAs were uploaded to GSEA v3.0 software.

### RNA expression analysis by cBioPortal

371 samples of HCC were prepared to determine the mRNA expressions of selected genes utilizing the cBioPortal database. An alteration analysis was conducted on mutation copy number and on the expression of selected gene. Clinical feathers such as overall survival and disease free survival were also analyzed by cBioPortal (http://www.cbioprtal.org/).

### Tissues samples

Tissue specimens were collected from 52 HCC patients at three different stages (28 cases of progressive stage, 11 cases of stable stage and 13 cases of remission stage). All patients were admitted to the First Affiliated Hospital of Zhengzhou University from March 2014 to March 2016 and were diagnosed by pathology. The middle part of the tumor and the adjacent normal liver tissue were collected from each specimen. HCC tissues and adjacent normal tissues were stored at − 80 °C in order to perform following quantitative real-time (qRT)-PCR and western blotting analysis. The Ethics Committee of the First Affiliated Hospital of Zhengzhou University approved this experiment. All tissue samples were obtained with informed consent from patients.

### Cell culture and animals

Human normal hepatocytes LO2 cells (ATCC, USA) and six HCC cell lines, including SNU-886 (ATCC, USA), HepG2, BEL7405, HCCC9810, SMMC-7721 and MHCC97H (BeNa Culture Collection, Jiangsu, China) were respectively cultured in DMEM medium (Gibco, USA) containing 10% fetal bovine serum, 100 U penicillin and 100 mg streptomycin in an incubator with 5% CO_2_ at 37 °C. Fresh medium was replaced every 2–3 days. The cells were digested with 0.25% Trypsin–EDTA when confluence reached approximately 80–90%.

9 SPF of BALB/c-nu mice were purchased from Animal Experimental Center of Zhejiang Academy of Medical Sciences (Zhejiang, China). Mice were raised in cages at room temperature (22 ± 3 °C) with a constant humidity (50 ± 10%) and provided with free access to food/water in a light/dark cycle (12 h/12 h). Animal experiments were performed according to the First Affiliated Hospital of Zhengzhou University Animal Ethics Committee and Guidelines for the Care and Use of Laboratory Animals.

### Cell transfection

Silent TRIP13 and empty control plasmids were purchased from Santa Cruz Biotechnology (USA). Two cell lines HepG2 and MHCC97H cells were selected for the transient transfection using Lipofectamine 2000 (Invitrogen, USA) according to manufacturer’s protocol. Two cells were respectively seeded in 6-well plate (1.0 × 10^5^) for 24 h before transfection. A total of silencing RNA, mock and Lipofectamine 2000 were respectively added to Opti-MEM medium and incubated at 25 °C for 20 min. Lipofectamine 2000 was then mixed into each well, which was cultured in Opti-MEM RPMI 1640 medium. After 6 h of culturing, the fluid was changed back to RPMI 1640 medium containing 10% FBS.

### Cell counting kit-8 (CCK-8)

Cell viability was measured by CCK-8 assay. The cells were transfected and respectively cultured for 12, 24 and 48 h, and 10 μl CCK-8 solution were added into each well and incubated for another 3 h at 37 °C. Cell viability was determined by recording the OD at a test wavelength 450 nm using a microplate reader (Thermo Fisher, USA).

### Flow cytometry

Cell apoptosis was performed on HepG2 and MHCC97H cells by flow cytometry. The cells were washed twice using washing buffer, and the suspensions were cultured with Annexin V-FITC and propidium iodide (Yeasen Biotechnology, Shanghai, China) in the dark at room temperature for 20 min. Next, binding buffer was added into each well. The samples were finally analyzed using FACS Calibur flow cytometer (BD Biosciences, San Jose, CA, USA) within 1 h.

### Transwell assay

Cell migration and invasion were performed by Transwell assay. After being transfected, HepG2 and MHCC97H cells were resuspended in serum-free medium. Upper chamber coated with matrigel was added into the cells. DMEM medium containing 10% fetal bovine serum was used in the lower 24-well chamber and the cells were incubated at 37 °C for 24 h. Next, the cells were fixed with 1% formaldehyde for 10 min at room temperature and then stained with 0.5% crystal violet for 5 min. Invaded cells were then counted at 200× magnification. Matrigel-coated was not used in migration assay and other steps were similar to that performed in the invasion assay.

### Quantitative real-time polymerase chain reaction (qRT-PCR)

TRIP13, Ki67, TIMP-2 and MMP-2 were detected by qRT-PCR in different groups. According to the manufacturer’s protocol, several RNAs were isolated from tissues or cultured cells by using Trizol (Invitrogen, USA). Reverse transcription was carried out at 37 °C for 15 min and at 85 °C for 5 s by OrimeScript ™ RT reagent kit (TaKaRa, Otsu, Shiga, Japan). cDNA amplified used SYBR Fast qPCR Mix (Invitrogen, USA). The conditions of cycles of TRIP13 and Ki67 were set as follows: a pre-denaturation at 95 °C for 5 min, followed by 40 cycles of denaturation at 95 °C for 15 s, annealing/elongation at 57 °C for 45 s. The condition of cycle of MMP-2 was set at 94 °C for 3 min, 32 cycles at 94 °C for 30 s, at 58 °C for 30 s; The condition of cycle of TIMP-2 was set at 94 °C for 3 min, 29 cycles at 94 °C for 30 s, at 57 °C for 30 s. GAPDH served as an internal control. All primers were used in synthesis (Sangon Biotech, Shanghai, China). The primer sequences were summarized in Table 1. Amplified products were electrophoresed through 2% agarose gels. The amount of RNA was calculated using the 2^−ΔΔCT^ method.

**Table 1:** Primers used in qRT-PCR

Gene	Primer	Sequence
TRIP13	Forward	5′-ACTGTTGCACTTCACATTTTCCA-3′
	Reverse	5′-TCGAGGAGATGGGATTTGACT-3′
Ki67	Forward	5′-CCATATGCCTGTGGAGTGGAA-3′
	Reverse	5′-CCACCCTTAGCGTGCTCTTGA-3′
TIMP-2	Forward	5′-GTGCCTCTGGATGGACTG-3′
	Reverse	5′-AGGAAGGGATGTCAGAGC-3′
MMP-2	Forward	5′-CACCTACACCAAGAACTTCC-3′
	Reverse	5′-AACACAGCCTTCTCCTCCTG-3′
GAPDH	Forward	5′-CGGAGTCAACGGATTTGGTCGTAT-3′
	Reverse	5′-AGCCTTCTCCATGGTGGTGAAGAC-3′

### Western blotting analysis

Proteins were extracted from tissues or cells using RIPA lysis buffer (Thermo Scientific, USA). Bradford method (Amresco, USA) was used to determine proteins concentration. Aliquots of supernatant containing proteins were mixed with loading buffer and the sample was subjected to 10% SDS-PAGE gel. The resolved proteins were then transferred to a piece of polyvinylidene difluoride membrane and the blots were blocked in 1% milk, TBS, 0.1% Tween-20. Proteins were incubated with primary antibodies as follows: rabbit anti-TRIP13 antibody (ab204331, 1:100, Abcam, USA), anti-Ki67 antibody (ab16667, 1:1000, Abcam, USA), anti-TIMP-2 antibody (ab180630, 1:500, Abcam, USA), anti-MMP-2 antibody (ab37150, 1:1000, Abcam, USA), anti-active-caspase3 antibody (ab2302, 1:1000, Abcam, USA), anti-TGF-β1 antibody (ab92486, 1:1000, Abcam, USA), anti-TβRII antibody (ab186838, 1:500, Abcam, USA), anti-smad3 antibody (ab40854, 1:1000, Abcam, USA), anti-p-smad3 antibody (ab63403, 1:1000, Abcam, USA) and anti-GAPDH antibody (ab9485, 1:2500, Abcam, USA) overnight at 4 °C. Following being washed 3 times in PBS, the blots were incubated with a rabbit whole molecule HRP- conjugated secondary antibody (Protein tech, 1:100,000, USA). Then the blots were washed with TBS four times for 5 min and the blots showed ECL (Thermo Fisher Scientific, Inc. USA). The quantification of the relative expression of protein was performed using Quantity one (Bio-Rad, USA).

### Xenograft experiment

HepG2 cells were transfected with silencing TRIP13 or mock plasmid. HCC model was induced by the hypodermic injection of 5 × 10^6^ cells into lateral abdominal wall of nude mice. Solid tumor formations in mice were set under the same raising condition about 21 days. Tumor volume and weight were measured every 3 days and the mice were sacrificed 49 days after cells transplantation.

### Immunohistochemical detection

Three groups of sections were collected from mice xenograft experiment. Sections were first deparaffinized in xylene after being placed at 60 °C overnight, and then dehydrated with gradient concentrations of ethanol and washed with 3% H_2_O_2_. Hot sodium chloride citrate buffer was used to renovate antigen for 20 min. The samples were incubated with TRIP13 antibody (ab204331, 1:100, Abcam, USA) at 4 °C overnight. The samples were washed by PBS and incubated at room temperature for 30 min with secondary antibody HRP-conjugated goat anti-Rabbit Ig G (Protein tech, USA). Diaminobenzidine (DAB) was performed as chromogen and hematoxylin was used to redye. Staining patterns were determined in selected representative slices. Staining intensity was categorized as negative, popcorn, yellow or brown.

### Terminal-deoxynucleotidyl transferase mediated nick end labeling (TUNEL) staining

The tumor tissues were treated with protease K for 15 min at 25 °C. Cell apoptosis was detected using in situ cell death detection kit (KeyGEN BioTECH, Jiangsu) following the manufacturer’s protocol and stained as brown. The results were observed through a microscope at 100× and 200× magnification.

### Statistical analysis

Statistical analysis was detected by Prism Graphpad version 6.0 software. All data were presented as mean ± standard deviation (SD). Differences were analyzed using one-way analysis of variance (ANOVA) following Turkey’s multiple comparison. A *P *< 0.05 was considered as statistically significant.

## Results

### Bioinformatics analysis of TRIP13

**Fig. 1:**
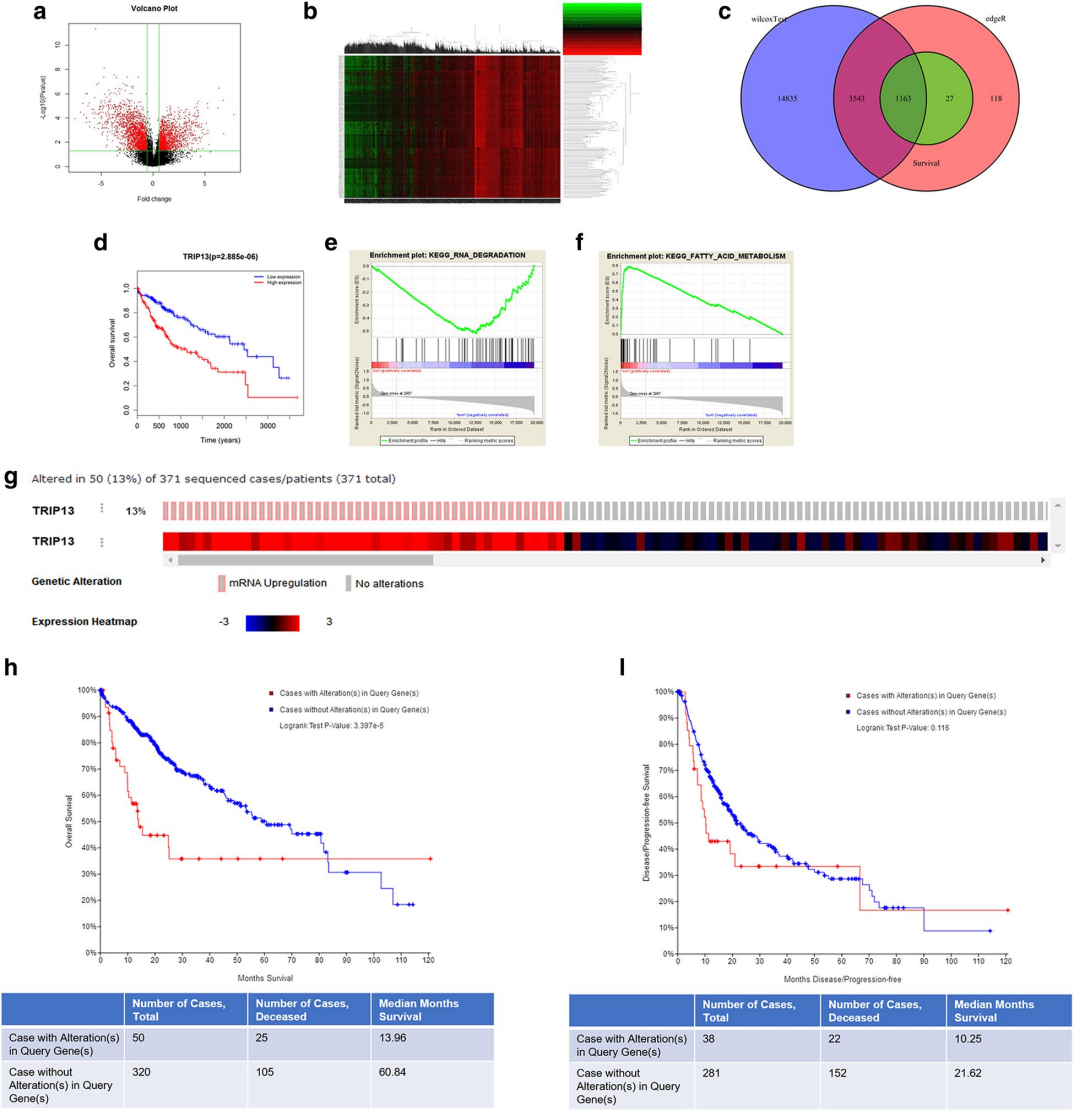
Bioinformatics analysis of TRIP13. **a** The visual hierarchical cluster analysis was performed using Volcano plot in HCC. **b** The visual hierarchical cluster analysis was performed using Heat map in HCC. **c** The cases of 50 normal liver tissues and 374 HCC tumor tissues were downloaded from TCGA. 1163 of cross differential genes were obtained from edgeR, WilcoxTest and survival analysis via Venn diagram. **d** The expression of TRIP13 with overall survival was analyzed by Kaplan–Meier curve. P = 2.885e−06. The biology significance was performed by GSEA on KEGG pathways. TRIP13 was mainly increased in RNA degradation (**e**) and fatty acid metabolism (**f**). **g** Transcriptional expression analysis of TRIP13 was shown by cBioPortal in HCC. mRNA analysis for HCC were available on cBioPortal with 371 samples. Survival analysis by TRIP13 mRNA expression was shown on cBioPortal in HCC. The overall survival (**h**) and the disease-free survival (**i**) were shown in months in the Kaplan–Meier analysis

The cases of 50 normal liver tissues and 374 HCC tumor tissues were downloaded from TCGA. 4851, 198541 and 1190 of DEGs were respectively analyzed from edgeR, WilcoxTest and survival analysis. 1163 of cross differential genes were obtained from the three softwares (Fig. 1c). TRIP13 high expression is associated with poor prognosis of patients with liver, breast, gastric and lung cancer [26]. The bioinformatics analysis of volcano plot, heat map and overall survival also predicted that TRIP13 was up-expression in HCC (Fig. 1a, b), and such a phenomenon was related with a poor survival rate (P = 2.885e−06, Fig. 1d). The biological significance was analyzed by GSEA according to KEGG pathways, and result showed that TRIP13 was mainly enriched in RNA degradation (Fig. 1e) and fatty acid metabolism (Fig. 1f). As Fig. 1g shown, an alteration of TRIP13 was described in 13% (50/371) of the samples. Especially, we found 50 up-regulated mRNA expressions of TRIP13 (Fig. 1g). The overall survival samples with altered TRIP13 pointed to a significantly low survival rate in comparison to the patients without alteration (P = 3.397e−5, Fig. 1h). For disease/progression-free, the result did not show a significant difference that there was 10.25 median months survival with alteration compared to 21.62 median months survival without alteration (P = 0.116, Fig. 1i). The result suggested that the overall survival of patients with high expression of TRIP13 analyzed from cBioPortal was similar to that of TCGA analysis.

### Up-regulated expressions of TRIP13 is associated with the progression of HCC and is in HCC cell lines

**Fig. 2:**
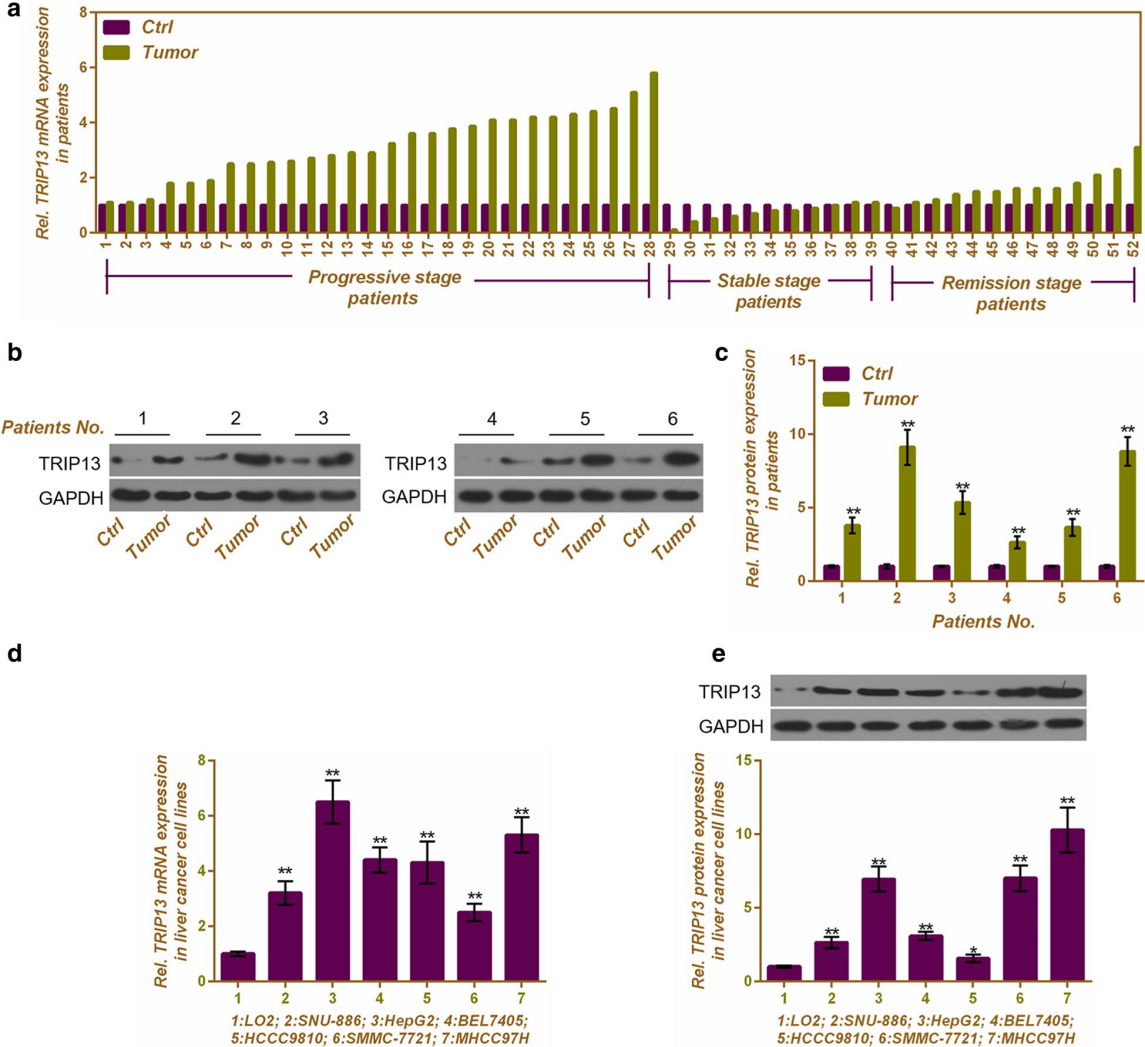
Up-regulated expression of TRIP13 is associated with the progression of HCC and is in HCC cell lines. **a** HCC patients with different stages were detected the mRNA expression of TRIP13 in its tumor tissue and adjacent normal tissue. **b** Six randomly selected HCC patients was detected the protein expression of TRIP13 in its tumor tissue and adjacent normal tissue. **c** Related TRIP13 expressions was showed as bar diagrams. GAPDH served as an internal control. Data were expressed as mean ± SD from three independent experiments (*compared with adjacent tissues, *P < 0.05, **P < 0.01). Normal hepatocytes LO2 cells and six HCC cell lines, including SNU-886, HepG2, BEL7405, HCCC9810, SMMC-7721 and MHCC97H were used to detect the mRNA (**d**) and protein expression (**e**) of TRIP13. GAPDH served as an internal control. Data were expressed as mean ± SD from three independent experiments (*compared with LO2 cells, *P < 0.05, **P < 0.01)

Tumor tissues and adjacent normal tissues from 52 HCC patients were collected, and the mRNA expression of TRIP13 was analyzed. As Fig. 2a shown, mRNA expression of TRIP13 was associated with the HCC progression. In detail, high expression of TRIP13 was obvious in progressive and remission stage of HCC. The mRNA level of TRIP13 in tumor tissues and adjacent normal tissues had no significant differences among HCC patients in stable stage (Fig. 2a). 6 HCC patients were selected randomly and analyzed the protein expression of TRIP13 by western blotting (Fig. 2b). The result showed that the protein level of TRIP13 was up-regulated in tumor tissues compared to normal tissues (P < 0.01, Fig. 2c). Normal hepatocytes LO2 cells and six HCC cell lines including SNU-886, HepG2, BEL7405, HCCC9810, SMMC-7721 and MHCC97H were determined to detect the mRNA and protein expressions of TRIP13. All HCC cell lines showed a high expression of TRIP13 in comparison to that of LO2 cells both at RNA and protein level (P < 0.01, Fig. 2d, e). Among six HCC cell lines, HepG2 and MHCC97H cell lines were identified to conduct the following experiments for the expression of TRIP13 was higher in HepG2 and MHCC97H cells than other cell lines.

### Silencing TRIP13 inhibits cell proliferation and migration and invasion, promotes cell apoptosis in HepG2 and MHCC97H cells

**Fig. 3:**
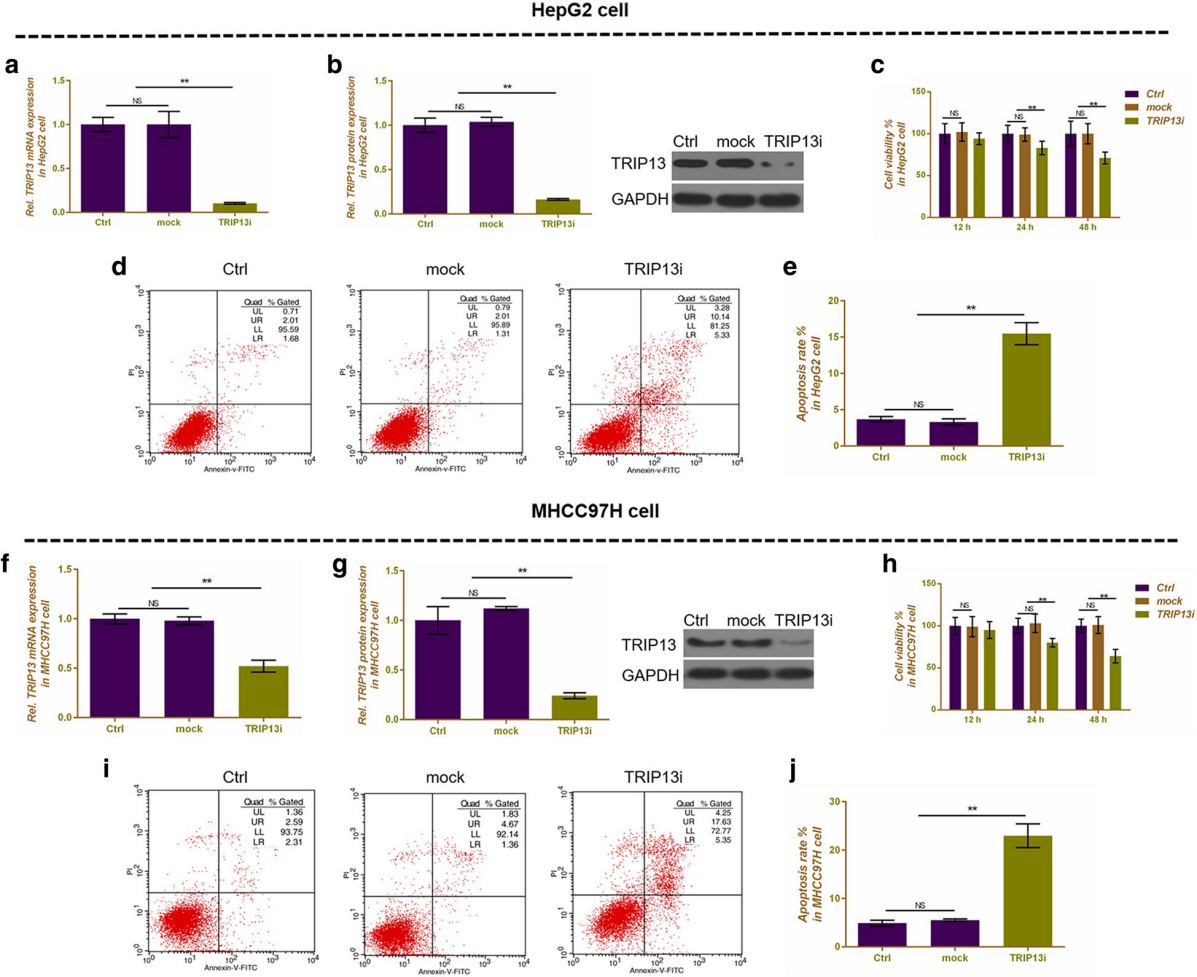
The effects of silencing TRIP13 on cell viability and cell apoptosis in HepG2 and MHCC97H cells. **a** Silencing TRIP13 decreased the mRNA expression of TRIP13, which was performed by qRT-PCR in HepG2 cells. **b** Silencing TRIP13 decreased the protein expression of TRIP13, which was performed by western blot in HepG2 cells. GAPDH served as an internal control. **c** Cell viability was performed by CCK-8 assay at 12, 24 and 48 h in HepG2 cells. **d** Flow cytometry was used to detect the effect of silencing TRIP13 on cell apoptosis in HepG2 cells. **e** Apoptosis rate was shown as bar diagrams in HepG2 cells. **f** Silencing TRIP13 decreased the mRNA expression of TRIP13, which was performed by qRT-PCR in MHCC97H cells. **g** Silencing TRIP13 decreased the protein expression of TRIP13, which was performed by western blot in MHCC97H cells. GAPDH served as an internal control. **h** Cell viability was performed by CCK-8 assay at 12, 24 and 48 h in MHCC97H cells. **i** Flow cytometry was used to detect the effect of silencing TRIP13 on cell apoptosis in MHCC97H cells. **j** Apoptosis rate was shown as bar diagrams in MHCC97H cells. Data were expressed as mean ± SD from three independent experiments (*compared with control or mock, *P < 0.05, **P < 0.01)

**Fig. 4:**
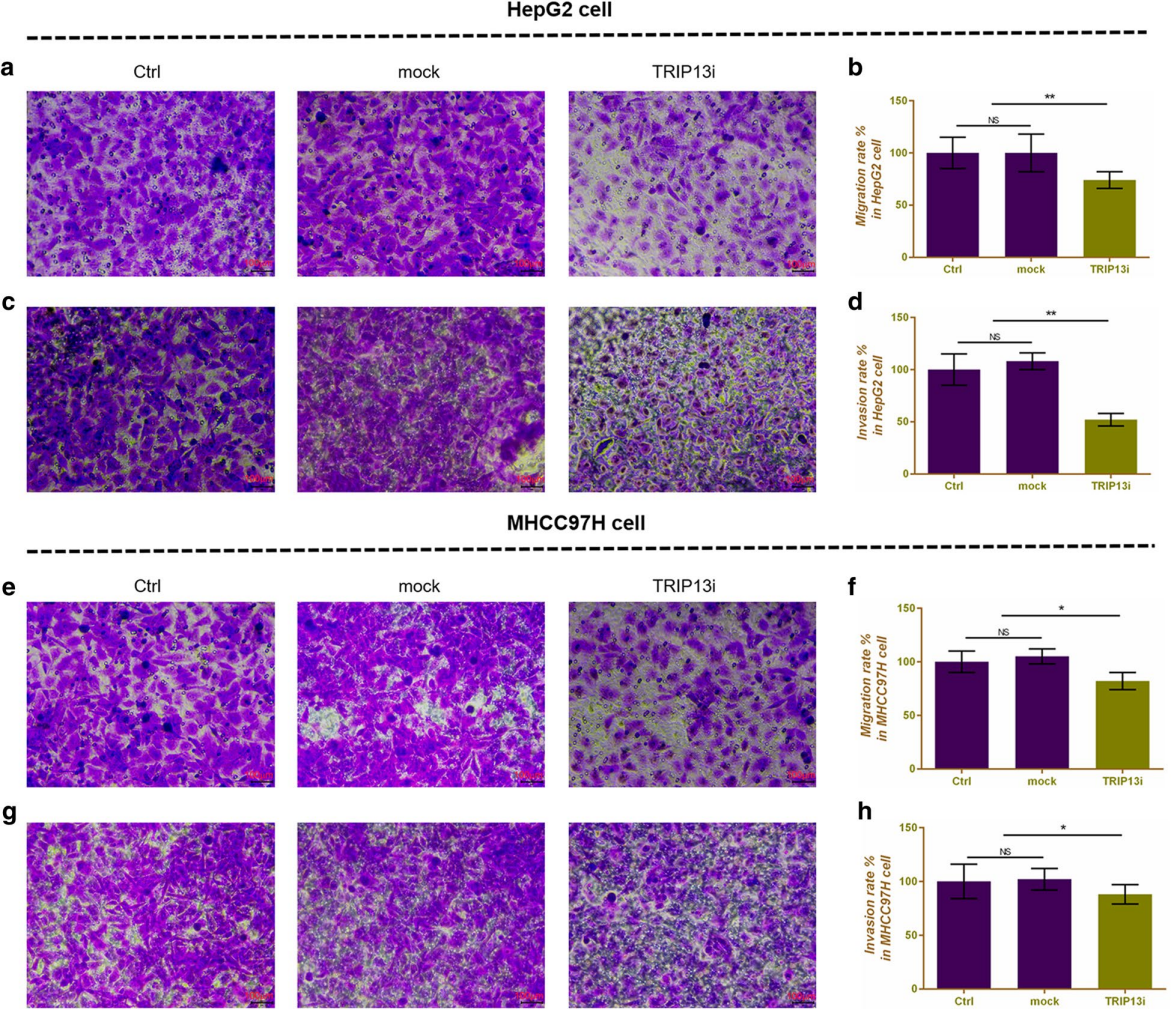
The effects of silencing TRIP13 on cell migration and invasion in HepG2 and MHCC97H cells. Transwell assay was performed to detect the effects of silencing TRIP13 on cell migration (**a**) and invasion (**c**) in HepG2 cells. Migration (**b**) and invasion (**d**) rate was shown as bar diagrams in HepG2 cells. Transwell assay was performed to detect the effects of silencing TRIP13 on cell migration (**e**) and invasion (**g**) in MHCC97H cells. Migration (**f**) and invasion (**h**) rate was shown as bar diagrams in MHCC97H cells. Data were expressed as mean ± SD from three independent experiments (*compared with control or mock, *P < 0.05, **P < 0.01)

Silencing TRIP13 was successfully transfected in HepG2 (Fig. 3a, b) and MHCC97H (Fig. 3f, g) cells, and both mRNA and protein expressions of TRIP13 were significantly down-regulated (P < 0.01). Cell viability was determined by CCK-8 assay, and as Fig. 3c, h shown, silencing TRIP13 could significantly inhibit cell viability at 24 h in both HepG2 and MHCC97H cells (P < 0.01, Fig. 3c, h). Cell apoptosis was determined using flow cytometry. In HepG2 cells, silencing TRIP13 noticeably increased apoptosis rate (15.47%), compared with control (3.69%) or mock (3.32%) (P < 0.01, Fig. 3d, e). Silencing TRIP13 had a similar effect of increasing (22.98%) apoptosis in MHCC97H cells, compared with control (4.90%) or mock (6.03%) (P < 0.01, Fig. 3i, j). Cell migration and invasion were detected by Transwell assay in HepG2 (Fig. 4a, c) and MHCC97H cells (Fig. 4e, g), and we found that cell migration and invasion were remarkably decreased by silent TRIP13 both in HepG2 cells (P < 0.01, Fig. 4b, d) and MHCC97H cells (P < 0.05, Fig. 4f, h) in comparison to control or mock.

### Silencing TRIP13 affects the expression of cell proliferation-, apoptosis-, migration and invasion-related genes in HepG2 and MHCC97H cells

**Fig. 5:**
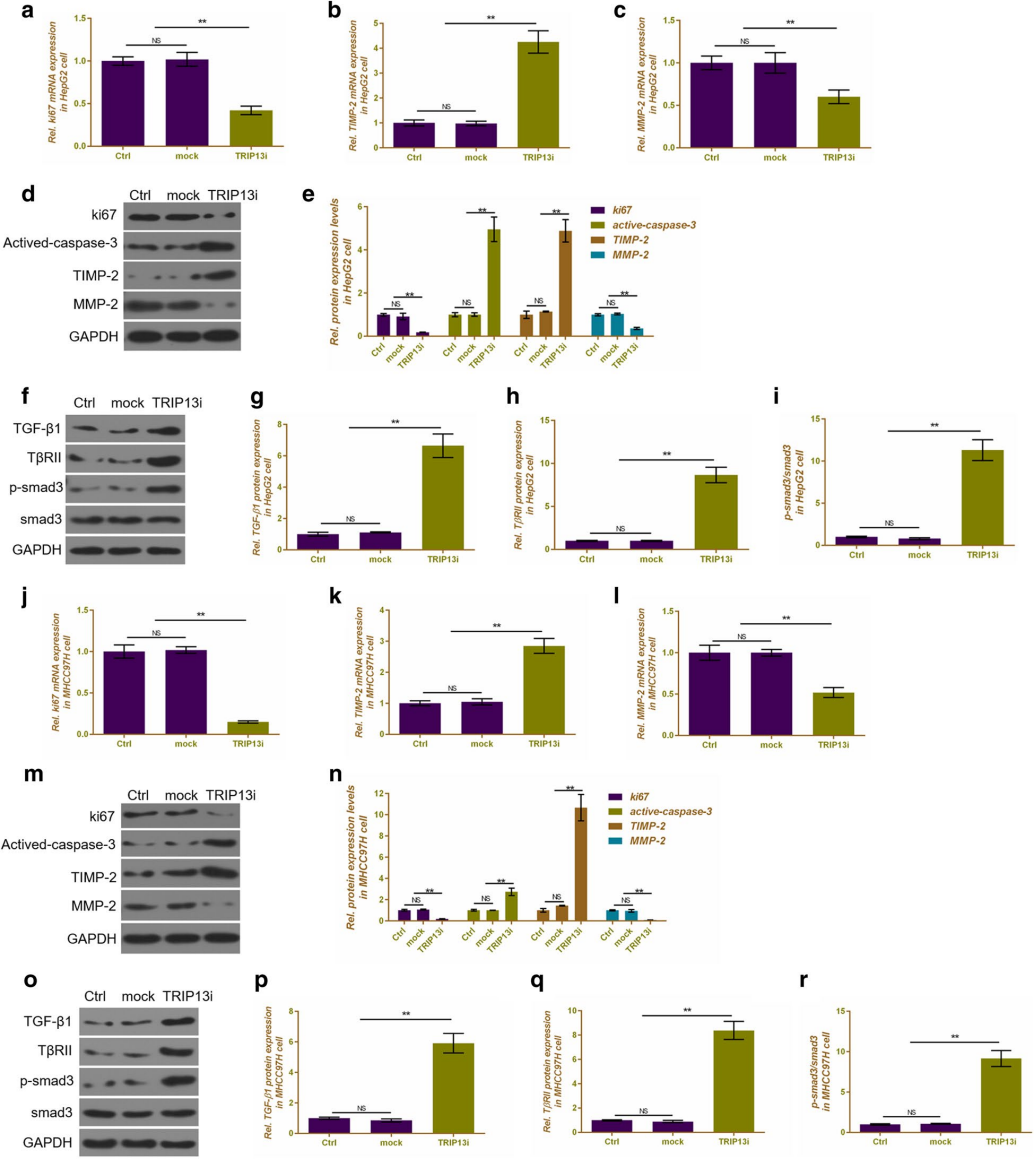
The effects of silencing TRIP13 on the expressions of related genes in HepG2 and MHCC97H cells. qRT-PCR was performed to detect the mRNA expressions of Ki67 (**a**), TIMP-2 (**b**) and MMP-2 (**c**) in HepG2 cells. **d** Western blot was performed to assess the protein expressions of Ki67, active-caspase-3, TIMP-2 and MMP-2 in HepG2 cells. **e** Relative protein levels of Ki67, active-caspase-3, TIMP-2 and MMP-2 were shown as bar diagrams in HepG2 cells. **f** Western blot was performed to assess the protein expressions of TGF-β1, TβRII, p-smad3 and smad3 in HepG2 cells. Relative protein expressions of TGF-β1 (**g**), TβRII (**h**) and p-smad3/smad3 (**i**) were shown as bar diagrams in HepG2 cells. qRT-PCR was performed to detect the mRNA expressions of Ki67 (**j**), TIMP-2 (**k**) and MMP-2 (**l**) in MHCC97H cells. **m** Western blot was performed to assess the protein expression of Ki67, active-caspase-3, TIMP-2 and MMP-2 in MHCC97H cells. **n** Relative protein levels of Ki67, active-caspase-3, TIMP-2 and MMP-2 were shown as bar diagrams in MHCC97H cells. **o** Western blot was performed to assess the protein expressions of TGF-β1, TβRII, p-smad3 and smad3 in MHCC97H cells. Relative protein expressions of TGF-β1 (**p**), TβRII (**q**) and p-smad3/smad3 (**r**) were shown as bar diagrams in MHCC97H cells. GAPDH served as an internal control. Data were expressed as mean ± SD from three independent experiments (*compared with control or mock, *P < 0.05, **P < 0.01)

We further detected the mRNA and protein expressions of related genes. In HepG2 cells, silencing TRIP13 not only decreased significantly decrease the mRNA and protein expressions of Ki67 (P < 0.01, Fig. 5a, d, e) and MMP-2 (P < 0.01, Fig. 5c–e), but also completely increased the mRNA and protein levels of TIMP-2 (P < 0.01, Fig. 5b, d, e) and up-regulated the protein expressions of active caspase-3 (P < 0.01, Fig. 5d, e). The regulated effects of silencing TRIP13 on MHCC97H cells were similar to that in HepG2 cells (P < 0.01, Fig. 5j–n).

### Silencing TRIP13 increases the related protein expressions of TGF-β1/smad3 pathway

Silencing TRIP13 significantly increased the protein expressions of TGF-β1 (P < 0.01, Fig. 5f, g), TβRII (P < 0.01, Fig. 5f, h) and p-smad3/smad3 (P < 0.01, Fig. 5f, i) in comparison to control or mock in HepG2 cells and MHCC97H cells (P < 0.01, Fig. 5o–r).

### Silencing TRIP13 inhibited the formation of HCC solid tumor in vivo

**Fig. 6:**
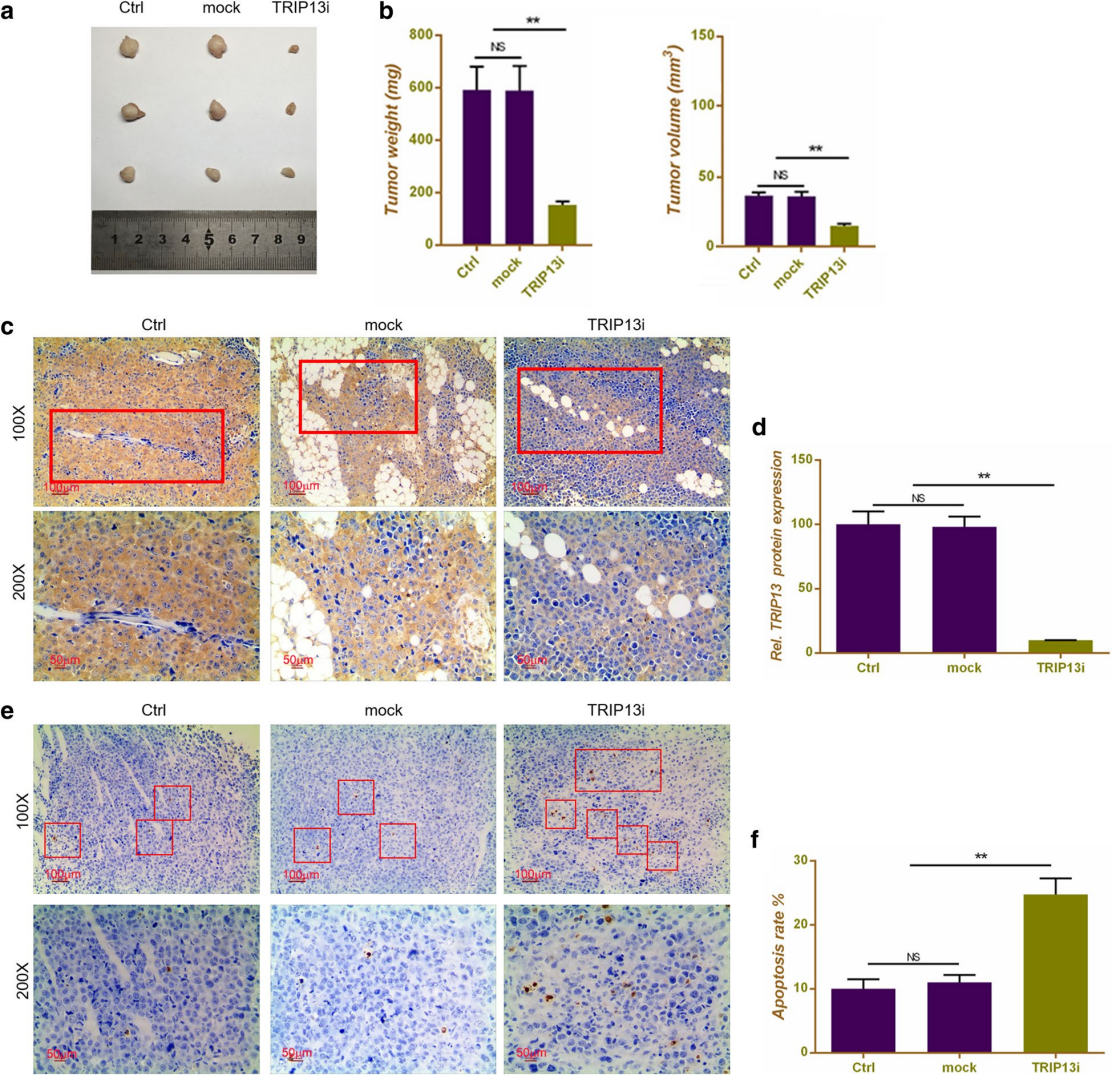
Silencing TRIP13 inhibited the formation of HCC solid tumor in vivo. **a** HCC tumor size comparison was shown in different groups, including control, mock and silencing TRIP13. **b** Quantitative analysis result of tumor weight and volume in mice transplanted with HepG2 cells. **c** Immunohistochemical detection of TRIP13 protein expression in mice tissues transplanted with HepG2 cells. **d** Quantitative analysis result of TRIP13 protein expression in mice tissues transplanted with HepG2 cells. Representative images (**e**) and quantitative analysis (**f**) results of TUNEL staining in mice tissues transplanted with HepG2 cells. Data were expressed as mean ± SD from three independent experiments (*compared with control or mock, *P < 0.05, **P < 0.01)

In order to explore the effect of silencing TRIP13 on tumorigenecity of Hepatocellular carcinoma, 9 nude mice were transplanted with HepG2 cells to establish HCC models in vivo. The results showed that tumor weight and volume had no clear difference in group control and mock. When mice were transplanted with HepG2 + silencing TRIP13, both weight and volume of tumor showed noticeable attenuations (P < 0.01, Fig. 6a, b). The IHC staining showed that both control and mock had a TRIP13 positive expression, and that tissues with silencing TRIP13 significantly down-regulated the protein level of TRIP13 (P < 0.01, Fig. 6c, d). TUNEL assay was performed to detect the apoptosis levels of tumor tissues. As Fig. 6e, f shown, silencing TRIP13 could remarkably increase the number of apoptosis cells in comparison to control or mock (P < 0.01, Fig. 6e, f), indicating that silencing TRIP13 inhibiting the formation of HCC solid tumor possibly involved cell apoptosis.

## Discussion

In the present study, we applied bioinformatics analysis to obtain 1163 genes of differential expression in HCC. Among the genes, TRIP13 draw our great interests as a high TRIP13 expression pointed to a poor prognosis of patients with various cancers including HCC [26]. TRIP13 was enriched in RNA degradation and fatty acid metabolism in KEGG pathway. Moreover, Up-regulated TRIP13 had a poor overall survival. Following experiments proved that TRIP13 was associated with the progression of HCC, and that a high expression of TRIP13 was mainly found in progressive and remission stage of tumor tissues and in several HCC cell lines.

Furthermore, silencing TRIP13 was successfully transfected into HCC cells. The findings showed that silencing TRIP13 could not only significantly inhibit cell viability, migration and invasion and induce cell apoptosis in vitro, but also inhibit the formation of tumor by increasing apoptosis level in vivo. In addition, related genes were also detected. Ki67 is an important tumor cell proliferation marker [31]. Our result found that silencing TRIP13 decreased the expression of Ki67 both at mRNA and protein level and such a phenomenon was in accordance with cell viability attenuation. In regard of cell migration and invasion related genes, the destruction of basement membrane by cancer cells is an important factor that leads to tumor growth, invasion and metastasis [32]. The main component of basement membrane is extracellular matrix (ECM). The imbalance of extracellular matrix environment will directly affect epithelial cells, leading to cell transformation and metastasis. Tumor growth requires a process of pre-existing barrier rupture and liver tissues remodeling, which are mainly regulated by matrix metalloproteinases (MMPs) and tissue inhibitors of matrix metalloproteinases (TIMPs) [33]. Overexpression of MMPs can erode the basement membrane barrier and promote cancer cell invasion [34]. TIMPs decrease the degradation of ECM by inhibiting the activity of MMPs [35]. We found that silencing TRIP13 could significantly decrease the expression of MMP-2 and increase the expression of TIMP-2. In addition, silencing TRIP13 also promoted the protein expression of active caspase-3, which is seen as an apoptotic executor [36].

**Fig. 7:**
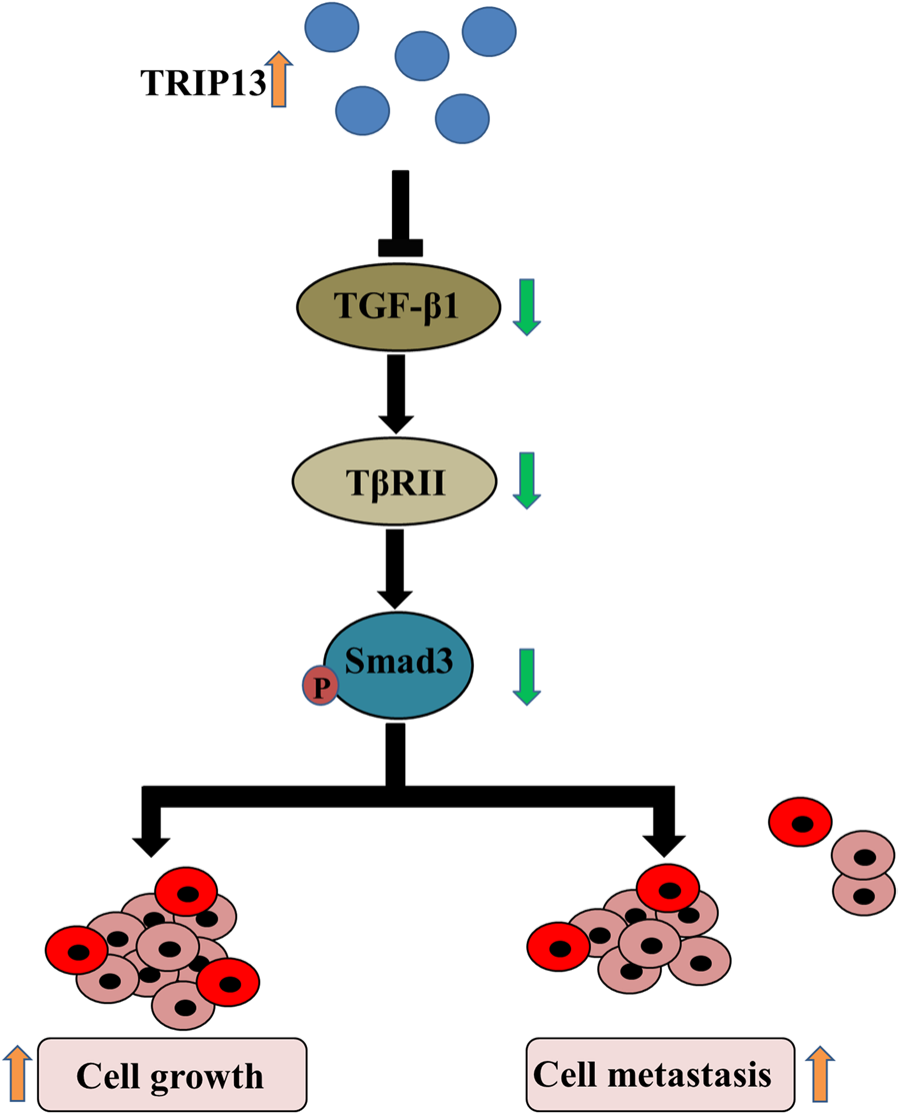
Schematic diagram of possible mechanism. TRIP13 expression could regulate hepatocellular carcinoma cell growth and metastasis via affecting the TGF-β1/smad3 signaling

In addition, we explored the underlying mechanism of silencing TRIP13 on inhibiting the growth and metastasis of HCC. TGF-β1 is a type of cytokine with many biological functions. By interfering with cell proliferation, differentiation, migration, invasion, attachment and ECM synthesis, TGF-β1 participates in various complicated physiological and pathological processes, for example, embryo development, inflammation, angiogenesis, fibrosis and carcinogenesis, in a human body [37–41]. The TGF-β1/smad signaling pathway has been proven to have a definite correlation to the treatment of hepatic fibrosis [42]. It can effectively treat liver fibrosis by reducing the activation and production of TGF-β1 and interfering with the expression of TGF-β1 downstream signaling pathways [43]. In this study, western blot was used to detect the expressions of important proteins of TGF-β1/smad signaling, including TGF-β1, TβRII and smad3. The findings showed that silencing TRIP13 could noticeably increase the proteins expressions of TGF-β1, TβRII and p-smad3/smad3 (Fig. 7), which is opposite to the results obtained in some previous studies [44–46]. For example, Yao reported that in keloids miR-1224-5p could promote cell apoptosis and decrease cell migration and invasion by inhibiting TGF-β1/smad3 [47]. Wang suggested that cryptotanshinone could ameliorate kidney fibrosis and epithelial transdifferentiation by inhibiting TGF-β1/smad3 [48]. However, the opposite result is reasonable as TGF-β has two sides in the process of HCC and TGF-β signaling plays a key role in inhibiting the progression of tumors [49, 50].

## Conclusions

Taken together, silencing TRIP13 acts as a tumor suppresser of HCC to inhibit cell proliferation, promote cell apoptosis and decrease cell migration and invasion in vitro and in vivo. The underlying mechanism may involve the activation of TGF-β1/smad3 signaling.
